# Engaging Parents With Child Nutrition and Feeding Information on Facebook: A Retrospective Content Analysis

**DOI:** 10.1002/fsn3.70326

**Published:** 2025-05-25

**Authors:** Maria Henström, Tayla Esplin, Emma Schwartzkoff, Kerith Duncanson, Gordana Popovic, Richard Ball

**Affiliations:** ^1^ Department of Medicine, Huddinge Karolinska Institutet Huddinge Sweden; ^2^ School of Health Sciences, College of Health, Medicine and Wellbeing University of Newcastle Callaghan New South Wales Australia; ^3^ University of New South Wales Sydney New South Wales Australia; ^4^ Health Promotion, Mid North Coast Local Health District Port Macquarie New South Wales Australia; ^5^ School of Medicine and Public Health, College of Health, Medicine and Wellbeing University of Newcastle Callaghan New South Wales Australia; ^6^ Food and Nutrition Research Program Hunter Medical Research Institute New Lambton Heights New South Wales Australia; ^7^ Stats Central, Mark Wainwright Analytical Centre University of New South Wales Sydney New South Wales Australia

**Keywords:** feeding behavior, food fussiness, internet‐based intervention, parents, social media

## Abstract

Online social media platforms are potentially useful for disseminating public health interventions, especially to parents who seek child nutrition information online. To optimize social media interventions, health professionals need to understand how to reach and engage the target audience. The PICNIC peer‐education nutrition program uses social media to teach parents about feeding practices which influence children's eating behaviors. The aim of this study was to describe social media post types, content, and communication strategies in the PICNIC program, and identify the characteristics that determined post performance. PICNIC Facebook Page intervention posts (*n* = 436) from Jan‐2020 to Apr‐2022 were evaluated using an iterative coding process with an adapted coding framework. Associations between coded post characteristics and organic reach (number of users) and user engagement (comments/shares/reactions/clicks) were explored using the Least Absolute Shrinkage and Selection Operator (LASSO) regression method in R. Photo posts reached more users than video posts, but videos drew 48% more silent engagement (clicks) than photos. Original content was associated with higher reach and more engagements than reported/shared posts. Post characteristics with positive influence on engagement included instructive (“how‐to”) communication techniques and feeding messages about food restriction and fussy eating. In conclusion, parents in an online child feeding program engaged more with social media posts that were instructive or in video format, with content relating to food restriction or fussy eating. Social media posts used in feeding interventions should include a variety of formats to optimize reach, balanced with engaging content useful to the specific target audience.

## Graphical abstract

A content analysis within the PICNIC peer‐education child‐feeding intervention showed that parents engaged more with social media posts that were instructive (“how‐to”) than informative, and content relating to food restriction or fussy eating. Social media‐based parent support interventions should include a variety of post formats to optimize reach, balanced with engaging content that resonates with the specific target audience.

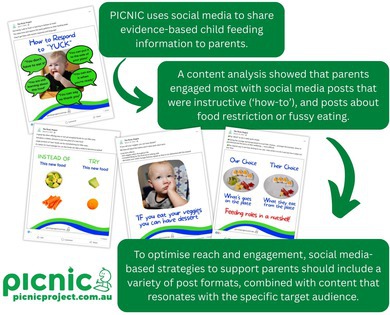

## 1 Introduction

Dietary intake in childhood is a strong predictor of child health and nutritional status (Juonala et al. 2013) and is linked to chronic disease prevention throughout life (Moore et al. 2016; Qi and Niu 2015). The Australian Dietary Guidelines recommend daily minimum food group servings for optimal child nutrition, based on age and sex (National Health and Medical Research Council 2013). By the age of two, the diet of Australian children already deviates from the recommendations, with only 6% of children aged 2–17 reaching the recommended intake of fruit and vegetables (Australian Institute of Health and Welfare 2018). Early eating behaviors, dietary patterns, and taste preferences are predominantly formed by 2–3 years (Skinner et al. 2002) and can persist into later life (Nicklaus et al. 2005).

Parental feeding practices are the behaviors associated with the provision of food to children and are pivotal determinants of a child's eating behaviors and food choices (Scaglioni et al. 2018; Ventura and Birch 2008). Many caregivers experience stress and anxiety surrounding parental feeding and child eating (Chan et al. 2011), with up to 50% of parents perceiving their preschool aged child to be a “picky/fussy eater” (Carruth et al. 2004; Dubois et al. 2007). Infants and young children are more likely to accept sweet flavors and reject sour or bitter flavors (Mennella and Bobowski 2015). This can inadvertently skew parents' food provision and child intake to more palatable easily acceptable foods, compromising food variety and nutrition quality (Savage et al. 2007).

Children's food preferences and consumption can be modified by their early life feeding experiences, such as repeated neutral food exposure to allow familiarity and acceptance (Wardle et al. 2003; Sullivan and Birch 1994, 1990). A neutral exposure means offering food without pressure or expectation to eat, and is considered effective in promoting intake of unfamiliar foods in children (Daniels 2019; Holley et al. 2017). Children exposed to a large variety of healthy foods during early weaning consume a more varied and quality diet throughout childhood (Cooke 2007). Studies show that up to 15 exposures may be needed for a food to become accepted (Birch et al. 1987). In practice, parents usually offer an unfamiliar food to their child less than three times before stopping and considering the food to be “disliked” (Carruth and Skinner 2000). Parents therefore need reinforcement and reassurance to continue with neutral food exposure until children accept unfamiliar foods.

PICNIC (Parents in Child Feeding Informing Community) aims to educate families about parental feeding practices (Ball et al. 2019). Informed by a previous pilot study (Ball et al. 2017), PICNIC was established on the Mid North Coast of New South Wales, Australia, in June 2018 and has been ongoing since then. The program provides training and support to new parents to on‐share infant/child feeding information and resources within their social networks. For the 12‐months intervention, parents of children up to 3 years of age have been continuously recruited through channels including health services, early childhood services, social media and by other parents, directing new participants to PICNIC through the “expression‐of‐interest” page on the project webpage (www.picnicproject.com.au/) (Ball et al. 2019). PICNIC utilizes multiple strategies to influence change including dietitian‐led workshops for participants, a website with feeding information and resources, a closed Facebook (Fb) group and public social media pages including Instagram and Fb. PICNIC uses social media, specifically Fb, because participatory action research shows it to be parents preferred method to share information (Ball et al. 2022). The program aims to distribute evidence‐based parental feeding information to program participants as well as parents outside of the study population (Ball et al. 2019, 2022). In a recent study we found that public PICNIC social media posts reach and engage a large number of users on Fb, but that reach and engagement vary greatly between posts (Henström et al. 2022). As a next step we proposed a content analysis to gain insights into what types of content and posts are more or less engaging to PICNIC users.

Parents obtain a large proportion of information about child health, child feeding and nutrition from social media (Moon et al. 2019). Although social media is a potential tool to disseminate evidence‐based content, health professionals often lack the resources and expertise to optimize reach and engagement (Probst and Peng 2019). Engagement is generally considered important for the effectiveness of an intervention (Short et al. 2018), due to its effect on reach (Newberry 2023), which allows information to be distributed to a larger audience. However, many aspects of the social media algorithms remain unknown, and therefore, content may affect reach independently from engagement (Newberry 2023; Lada et al. 2021). Additionally, there are different forms of engagement: “active” (post stories such as likes, comments, and shares) and “silent” (post consumptions such as video clicks‐to‐play and clicks on photos or links). Since active and silent engagement may influence reach differently, both should be explored. Scientific literature exploring reach and engagement of child feeding content on social media is scarce.

The aim of this study were to (1) describe PICNIC social media posts with regards to post types, child feeding content, and communication strategies; and (2) identify post characteristics that influence post performance in terms of organic (non‐paid) reach and engagement. Findings from this study will inform future social media post strategies in PICNIC and similar child feeding and health promotion initiatives to improve reach and engagement with parents.

## 2 Methods

### 2.1 Study Design

This retrospective audit study involved a content analysis of public Fb posts posted by the PICNIC team as part of an ongoing program (full protocol by Ball et al. 2019) and built on previous evaluation of the overall reach and engagement of the online PICNIC components (Henström et al. 2022). The PICNIC program uses a public Fb page as a communication channel to share evidence‐based child nutrition and feeding information to PICNIC parents (most being first‐time mothers) as well as other parents within their social networks. All content posted on the public PICNIC Fb page is also shared in the closed Fb group intended for parents enrolled in the program (Ball et al. 2022). This study is reported using the STROBE checklist for observational studies (File S1) (von Elm et al. 2007).

### 2.2 Data Extraction

**FIGURE 1:**
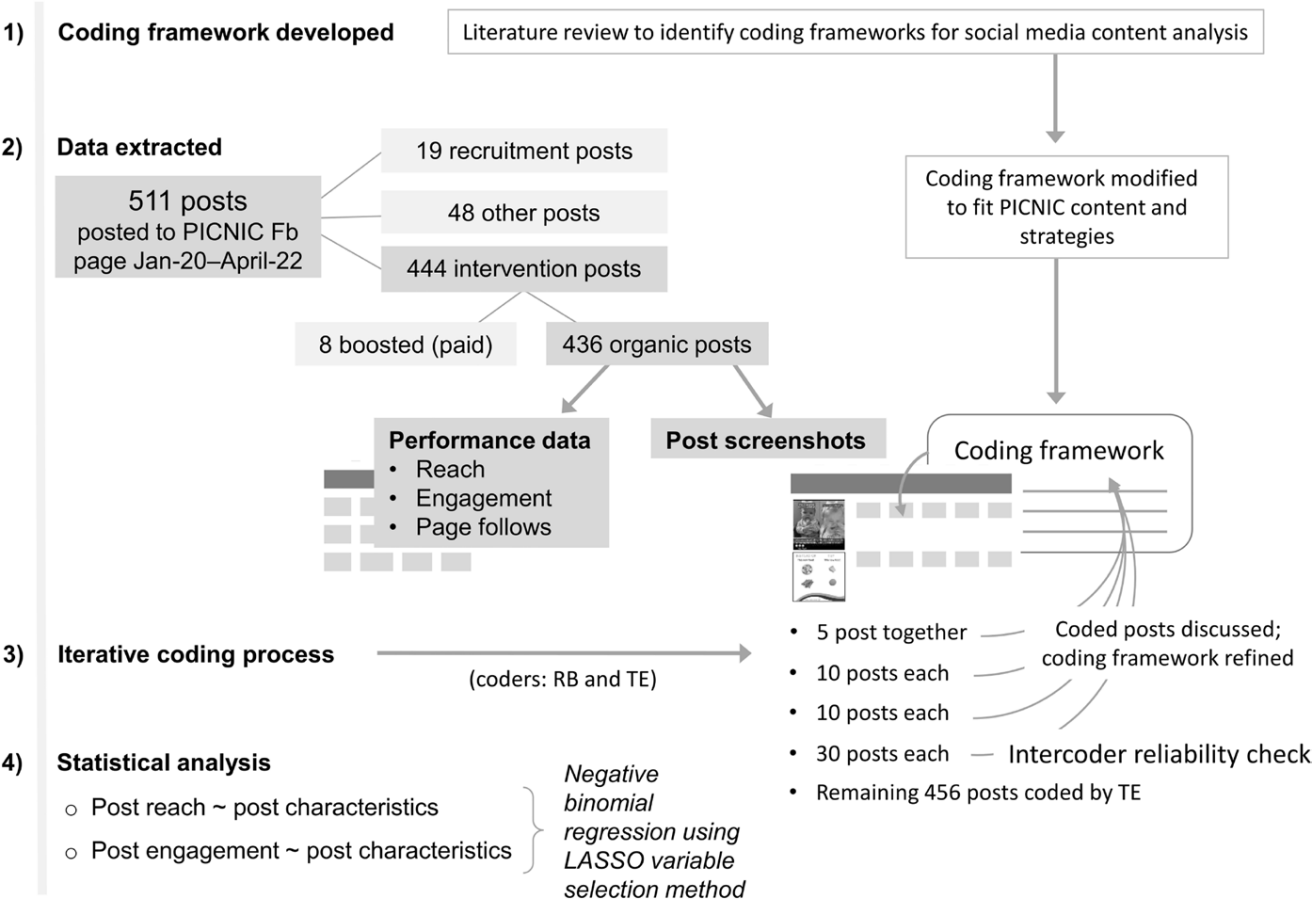
Flowchart of methodological process. Flowchart explaining the methodological process from development of the coding framework through to the statistical analysis. T.E. is a medical undergraduate student, R.B. is an Accredited Practising Dietitian, PhD student and principal investigator of PICNIC. In the iterative coding process, the coding framework was made clearer and rules were put into place to ensure more consistent coding, such as prioritizing the message in the photo over the message of the caption (see details in File S2). Any uncertainties were discussed with R.B., M.H., and E.S. to reach consensus.

Fb post‐performance data was exported from Fb Page Insights in early 2022, as shown in Figure 1. This data includes total reach and user engagement of posts on the public PICNIC Fb page, including that of further shared posts (e.g., in the PICNIC Fb group). The total number of PICNIC page followers on the date of posting were also exported. The final sample included all PICNIC page posts from the beginning of January 2020 until the end of April 2022 (28 months, 4–5 Fb posts/week). All PICNIC posts were available to Fb users for at least 1 month before data were extracted. Posts that received a paid “boost” were not included in the analyses, as the potential influence from higher (non‐organic) reach on user engagement could not be determined. A separate file with screenshots of all Fb posts (or link to videos) was prepared by a research assistant not involved in data analysis to ensure blinding when possible from the performance of Fb posts during coding.

### 2.3 Coding Framework and Process

A coding framework was developed to identify PICNIC Fb post characteristics and content strategies (described in File S2). The codes were based on frameworks used in previous research for social media content analyses (Kite et al. 2019, 2016; Klassen et al. 2018; Barklamb et al. 2020), but modified to match PICNIC content and strategies. This was done by triangulation of information from the previous frameworks, with responsive feeding constructs relevant to the PICNIC intervention target behaviors, in addition to other content types usually present in PICNIC posts. Thus, while categories such as format, links, prompts, communication technique, emotion‐inducing, and real‐world tie‐ins were similar to previous frameworks, age groups and feeding construct message categories were newly created and specific to this study and our target group. This study focused on intervention Fb posts with a clear child feeding message intended to reach PICNIC parents and their peers (i.e., family members, parent groups, or friends with children, who were either following the PICNIC Fb page or were able to see PICNIC content through viral reach). Example screenshots of PICNIC intervention posts from this period are shown in File S3. Other Fb posts (such as program recruitment posts) were excluded from analysis. The coding process (Figure 1) was iterative and included initial coding of 55 posts by two investigators (T.E. and R.B.). The intercoder reliability check was performed using kappa statistics (O'Connor and Joffe 2020). The overall kappa was 0.86, which indicates nearly perfect agreement between the two raters. Two‐thirds of the categories had nearly perfect agreement (kappa > 0.8) and the rest had substantial agreement (kappa between 0.6 and 0.8). The latter were discussed, and the coding framework was refined when required before the remaining posts were coded by T.E.

### 2.4 Outcome Measures: Reach and Engagement Metrics

Data included reach and user engagement of the posts. Reach refers to the estimated number of unique Fb users who had the post entering their screen at least once. Active engagement refers to the sum of post stories, that is, reactions (also known as post “likes”), comments, and shares. Some users may consume posts without actively engaging in it; this “silent” engagement refers to post clicks on a photo, a link, “See more” to expand the caption, video click‐to‐plays, or other post clicks. Total engagement was defined as the sum of both “active” and “silent” post engagement. Additional metrics exported for video posts included the number of auto‐played and clicked‐to‐play views, and views to at least 95% of the total video length.

### 2.5 Data Analysis

Statistical analysis was performed using R Statistical Software (R v.4.2.1, R Core Team, Vienna, Austria, 2022) and RStudio (v2022.2.3.492, RStudio Team, Boston, MA, 2022). Descriptive statistics provided an overview of PICNIC post characteristics and performance. Reach and engagement data were right skewed and therefore presented as median and interquartile range (IQR). Subcategories with few posts were merged with similar categories when appropriate or excluded in the final analysis (File S2).

User engagement with a Fb post will typically “feed” the algorithm to show the post to more users (i.e., increase the reach) (Newberry 2023), and post characteristics may influence reach, therefore we assumed reach is a “collider” and should not be controlled for when investigating the relationship between engagement and post characteristics. Conversely, when modeling reach as the outcome, we controlled for engagement, assuming it to be a confounder. To choose an appropriate distribution for each outcome, we inspected residuals from Gaussian, Poisson, and negative binomial regressions. The data were best modeled by a negative binomial distribution.

Our study aim was to investigate what post characteristics or content strategies may help predict reach and/or user engagement. To achieve this, Least Absolute Shrinkage and Selection Operator (LASSO) regression was used, with a negative binomial distribution. A major advantage with LASSO is it uses a regularization technique to avoid overfitting of data by introducing a “penalty term” to the least squares method, causing shrinkage of the estimated coefficients (Friedman et al. 2010). This retains the most important predictors in the model while others are excluded (coefficients being sent to zero). Through cross‐validation, an optimal shrinkage parameter “lambda” (a constant that is “tuning” the penalty) is selected, resulting in the least prediction error. In the present study, LASSO was used as a variable selection method; thus, the entire dataset was used to fit the model and find the optimal lambda. Five‐fold cross validation and the *set.seed* function were used in R for reproducible results. Page followers at time of posting were included in all analyses to account for the growth of PICNIC over time.

The first association tested was between reach and post characteristics that might have had a direct influence on reach because of the Fb algorithm (Newberry 2023). In this study, these were: format type, origin of post, links, and prompts. Their effects on reach were assessed independent of user engagement by including total engagement as a covariate in the model. Next, the influence of post characteristics on user engagement was investigated by fitting three models, one for each dependent variable (total, active and silent engagement), including all post characteristics as predictor variables. The categories with the largest number of posts were set as reference categories, except for “Emotion‐inducing” where “Positive” was used as reference. The R package “mpath” (Wang 2022) and the *cv.glmregNB* function were used to run LASSO, and results were presented as incidence rate ratios (IRRs). R script from the LASSO analyses is available as File S4. Finally, we explored viewing metrics for video posts originally created by PICNIC to understand more about users' engagement in videos. For this, descriptive statistics were used instead of regression analyses because of the few original PICNIC videos posted on Fb during this period (*n* = 32).

## 3 Results

During the assessed period, a total of 511 posts (4–5 per week) were posted on the public PICNIC Fb page. During this time, the PICNIC audience grew from 1123 to 2067 Fb page followers. Of the identified posts, 444 were intervention posts, 19 were for recruitment, and 48 were other types of posts. Eight posts received a paid “boost,” and these were excluded from analysis due to potential influence from the higher (paid) reach on user engagement. The final sample consisted of 436 organic intervention posts that were coded and analyzed. There was no missing data in the final dataset.

### 3.1 Description of PICNIC Intervention Posts

The median reach and engagement metrics for each post characteristic are presented in Table 1. Overall, PICNIC's Fb posts reached an average of 467 users (IQR 351–679). On average, 24 users engaged with each post, with about half being silent engagements and half active engagements. Most of the active engagements were likes. The most common format type was photos (89.2%) followed by videos (7.8%). Most posts were original (88.3%), meaning they were produced and posted directly by the PICNIC project team. The remainder were reposted or shared content. In 17.7% of posts, a clickable link was included that aimed to direct users to PICNIC's website for more information. Informative communication technique (i.e., educational types of posts providing facts and information describing child feeding and its' consequences) was slightly more often used (38.1% of posts) than instructive communication technique (i.e., “how‐to” types of posts with concrete tips and advice; 34.6%) and storytelling (i.e., real‐life examples, anecdotes or quotes; 27.3%), see File S2 for detailed code definitions. Around two‐thirds of posts utilized some emotion‐inducing tone, whilst the rest were either neutral in tone or unspecified. Certain feeding message topics were more frequently communicated than others. For instance, pressure to eat (29.6%) and food exposure (31.4%) were the most common post topics, while food rewards (3.4%), external influences (3.4%) and fussy eating (4.6%) were less common. We observed a strong positive correlation between active engagement and organic post reach (Spearman correlation; *r* = 0.75), and a moderate positive correlation between silent engagement and organic post reach (*r* = 0.51).

**TABLE 1:** Reach and engagement metrics by post characteristics for PICNIC Facebook posts.

		*n* (%)	Median and interquartile range (25th;75th percentile)						
			Reach	Engagement					
			Organic reach	Total engagement	“Silent” engagement (consumptions)	“Active” engagement (post stories)			
						Total “active” engagement	Likes	Comments	Shares
	All posts	436	467 (351;679)	24 (14;43)	12 (5;28)	11 (7;18)	9 (5;14)	0 (0;1)	2 (1;3)
Format type	Photo	389 (89.2)	484 (361;681)	23 (13;40)	10 (4;23)	12 (7;18)	9 (5:14)	0 (0;1)	2 (1;3)
	Video	34 (7.8)	351 (239;535)	45 (25;64)	34 (21;46)	9 (7;18)	7 (4;12)	0 (0;2)	2 (1;4)
	Other^a^	13 (3.0)	426 (232;599)	24 (6;91)	20 (2;72)	7 (2;14)	6 (2;11)	0 (0;1)	0 (0;1)
Origin of post	Original	385 (88.3)	486 (359;682)	24 (15;46)	12 (5;28)	12 (7;19)	10 (6;14)	0 (0;2)	2 (1;3)
	Reposted/shared	51 (11.7)	371 (305;539)	19 (11;33)	13 (6;23)	5 (3;10)	5 (3;9)	0 (0;1)	1 (0;1)
Links	PICNIC website	77 (17.7)	383 (290;513)	19 (12;33)	10 (5;18)	9 (5;14)	7 (4;11)	0 (0;1)	1 (1;3)
	Other links^a^	30 (6.9)	456 (324;726)	23 (12;68)	16 (6;42)	8 (5;17)	8 (3;11)	1 (0;2)	2 (1;3)
	No links	329 (75.5)	494 (362;687)	25 (15;46)	12 (5;29)	12 (7;18)	10 (6;14)	0 (0;1)	2 (1;3)
Prompting engagement	Prompt^a^	112 (25.7)	384 (301;561)	21 (12;40)	10 (6;26)	9 (5;16)	7 (4;12)	0 (0;1)	1 (1;3)
	No prompts	324 (74.3)	495 (367;696)	25 (15;45)	13 (5;30)	12 (7;18)	10 (6;14)	0 (0;1)	2 (1;3)
Communication technique	Informative	166 (38.1)	442 (349;672)	20 (12;35)	9 (4;18)	11 (6;16)	9 (5;13)	0 (0;1)	2 (1;3)
	Instructive	151 (34.6)	522 (390;726)	29 (17;49)	14 (5;32)	14 (9;20)	10 (7;15)	0 (0;2)	2 (1;4)
	Storytelling	119 (27.3)	424 (322;643)	24 (14;48)	15 (6;33)	9 (6;18)	8 (5;13)	0 (0;1)	2 (1;3)
Answer to question	Yes	87 (20.0)	454 (357;679)	23 (15;40)	12 (5;24)	11 (7;17)	9 (5;13)	0 (0;1)	2 (1;3)
	No	349 (80.0)	469 (349;675)	24 (14;45)	12 (5;28)	11 (7;18)	9 (5;14)	0 (0;1)	2 (1;3)
Emotion‐inducing	Positive	67 (15.4)	444 (364;574)	22 (14;44)	12 (4;33)	13 (7;18)	10 (5;14)	0 (0;1)	2 (1;3)
	Avoid/Negative	52 (11.9)	451 (347;682)	19 (13;39)	10 (5;22)	10 (5;16)	8 (4;13)	0 (0;1)	2 (1;3)
	Supportive	81 (18.6)	431 (323;647)	22 (12;37)	11 (4;20)	9 (6;16)	7 (5;12)	0 (0;1)	2 (1;3)
	Humorous	97 (22.2)	488 (356;679)	27 (15;45)	14 (6;27)	11 (7;18)	9 (5;14)	0 (0;2)	1 (1;3)
	Neutral/Other	139 (31.9)	497 (364;738)	27 (16;50)	13 (5;33)	13 (8;20)	10 (7;16)	0 (0;2)	2 (1;4)
Real‐world tie‐ins	People/Events/Culture	13 (3.0)	508 (418;767)	16 (13;34)	9 (5;15)	8 (7;13)	8 (6;12)	0 (0;0)	2 (1;2)
	None	423 (97.0)	463 (349;678)	24 (14;44)	12 (5;28)	11 (7;18)	9 (5;14)	0 (0;1)	2 (1;3)
Age group specific	6–12 months	48 (11.0)	401 (336;546)	26 (14;45)	17 (4;30)	13 (8;18)	10 (6;12)	0 (0;2)	2 (1;4)
	12+ months	100 (22.9)	536 (362;773)	29 (19;45)	14 (6;28)	14 (7;22)	11 (6;16)	0 (0;1)	2 (1;4)
	Both	288 (66.1)	459 (349;657)	23 (13;41)	11 (5;28)	10 (7;17)	8 (5;14)	0 (0;1)	2 (1;3)
Feeding message	*Learn to eat*								
	Yes	86 (19.7)	484 (350;693)	27 (16;45)	13 (5;30)	13 (7;18)	10 (6;14)	0 (0;2)	2 (1;3)
	No	350 (80.3)	463 (353;670)	24 (13;43)	12 (5;27)	11 (7;18)	9 (5;14)	0 (0;1)	2 (1;3)
	*Pressure to eat*								
	Yes	129 (29.6)	486 (349;682)	27 (16;45)	14 (6;28)	13 (7;18)	9 (6;14)	0 (0;2)	2 (1;3)
	No	307 (70.4)	460 (353;673)	23 (14;43)	11 (4;28)	10 (7;18)	9 (5;14)	0 (0;1)	2 (1;3)
	*Food restriction*								
	Yes	55 (12.6)	485 (377;786)	33 (14;49)	18 (6;32)	14 (8;21)	11 (7;17)	0 (0;2)	2 (1;4)
	No	381 (87.4)	463 (349;671)	24 (14;42)	12 (5;27)	11 (7;18)	9 (5;14)	0 (0;1)	2 (1;3)
	*Food exposure*								
	Yes	137 (31.4)	526 (385;734)	25 (17;42)	12 (5;26)	14 (8;19)	11 (7;15)	0 (0;1)	2 (1;3)
	No	299 (68.6)	449 (339;650)	23 (12;44)	12 (5;28)	10 (6;18)	8 (5;13)	0 (0;1)	2 (1;3)
	*Meal structure*								
	Yes	25 (5.7)	524 (367;767)	33 (19;51)	14 (6;39)	14 (9;25)	12 (7;20)	0 (0;1)	2 (1;3)
	No	411 (94.3)	463 (349;676)	24 (14;42)	12 (5;28)	11 (7;18)	9 (5;14)	0 (0;1)	2 (1;3)
	*Meal environment*								
	Yes	61 (14.0)	426 (349;548)	19 (9;34)	6 (3;15)	10 (6;16)	8 (5;14)	0 (0;1)	2 (1;3)
	No	375 (86.0)	486 (356;682)	25 (15;46)	13 (5;30)	12 (7;18)	9 (5;14)	0 (0;2)	2 (1;3)
	*Food rewards*								
	Yes	15 (3.4)	636 (409;756)	28 (23;45)	17 (11;31)	14 (9;17)	9 (6;14)	1 (0;3)	2 (1;3)
	No	421 (96.6)	463 (349;675)	24 (14;43)	12 (5;28)	11 (7;18)	9 (5;14)	0 (0;1)	2 (1;3)
	*Fussy eating*								
	Yes	20 (4.6)	497 (342;611)	24 (19;60)	16 (9;40)	13 (7;22)	10 (5;16)	0 (0;2)	2 (1;3)
	No	416 (95.4)	467 (353;680)	24 (14;42)	12 (5;28)	11 (7;18)	9 (5;14)	0 (0;1)	2 (1;3)
	*External influences*								
	Yes	15 (3.4)	411 (364;628)	16 (10;31)	6 (5;17)	9 (5;14)	7 (5;11)	0 (0;1)	1 (1;3)
	No	421 (96.6)	470 (349;679)	24 (14;44)	12 (5;28)	11 (7;18)	9 (5;14)	0 (0;1)	2 (1;3)
Type of message	*Recipes/food hacks*								
	Yes	73 (16.7)	513 (369;720)	31 (19;47)	14 (6;33)	14 (8;19)	10 (7;15)	0 (0;2)	2 (1;3)
	No	363 (83.3)	460 (349;671)	23 (14;42)	11 (5;27)	11 (6;18)	9 (5;14)	0 (0;1)	2 (1;3)
	*Child nutrition*								
	Yes	27 (6.2)	497 (362;644)	27 (19;52)	14 (7;36)	10 (9;17)	9 (6;14)	0 (0;1)	2 (1;3)
	No	409 (93.8)	463 (349;679)	24 (14;43)	12 (5;28)	11 (7;18)	9 (5;14)	0 (0;1)	2 (1;3)
	*Child health related*								
	Yes	22 (5.0)	365 (344;494)	24 (12;42)	16 (5;24)	8 (8;14)	7 (6;10)	0 (0;1)	2 (1;2)
	No	414 (95.0)	472 (353;679)	24 (14;43)	12 (5;28)	12 (7;18)	9 (5;14)	0 (0;1)	2 (1;3)

*Note:* Table 1 shows the reach and engagement metrics for each post characteristic of analyzed PICNIC Fb posts. PICNIC website: https://www.picnicproject.com.au/.

Abbreviations: *n*, number of posts; PICNIC, Parents in Child Nutrition Informing Community.

^a^ Merged categories original numbers: format type, other (merged Link posts (*n* = 10) and Text‐only posts (*n* = 3)); links, other links (merged external (*n* = 20) and social media (*n* = 10)); prompting engagement, prompts (merged requires an answer (*n* = 22), requires an answer (*n* = 90) and polls/quizzes (*n* = 0)). Silent engagement: consumptions, that is, any click on the post. Total active engagement: sum of all post stories, that is, reactions, comments, and shares.

### 3.2 Post Characteristics Predicting Reach

When investigating the influence of post characteristics (format type, origin of post, links, and prompts) on post reach, we included total engagement as a predictor in the model to be able to assess the effect on reach independent of user engagement levels. The strategy with the largest impact on post reach was format type; compared to photos, videos were predicted to receive 43% less reach (Table 2). Furthermore, a reposted/shared post was associated with 12% lower reach compared to originally created posts. Clickable links to the PICNIC website did not influence post reach compared to posts without links. Finally, although prompting engagement (such as asking participants to comment) was kept in the model after LASSO regression, the predicted effect on reach was small (7% lower than posts with no prompts).

**TABLE 2:** PICNIC Facebook posts (*n* = 436) characteristics predicting reach.

Predictor (number of posts)	IRR
**Format type**	
Photo (389)	Ref.
Video (34)	0.57
Other (13)	0.97
**Origin of post**	
Original (385)	Ref.
Reposted/shared (51)	0.88
**Links to health information**	
PICNIC website (77)	1.00
Other links (30)	1.05
No links (329)	Ref.
**Prompting engagement**	
Prompt (112)	0.93
No prompts (324)	Ref.

*Note:* LASSO results for the negative binomial regression model predicting the effects of content strategies on the reach of PICNIC Fb posts. The model is adjusted for PICNIC growth over time and post engagement by including the number of Fb page followers as well as total engagement (log transformed) as predictors in the regression model. The best log(lambda) chosen by LASSO and used in the regression was −2.59. Overall categories are in bold while subcategories are below. None of the included post characteristics predictor variables were excluded from the model after LASSO regression.

Abbreviations: IRR, incidence rate ratio; Ref., reference category.

### 3.3 Post Characteristics Predicting Engagement

The relationship between post characteristics and total, active, and silent engagement in PICNIC posts was investigated. The categories which remained after LASSO regression are shown in Table 3. Based on the results, post characteristics with the greatest positive influence on total engagement were video format type, original posts, instructive communication technique, and the feeding topics “food restriction” and “fussy eating.” Video posts positively influenced total engagement compared to photos. However, this effect was due to silent engagement only, which was 48% higher compared to photo posts in this model. Reposted or shared content received less engagement compared to original content produced by PICNIC. This was most evident for active engagement with 51% lower active engagement compared to 16% lower silent engagement. Instructive posts, which focused on giving advice to parents using illustrative “how‐to” messages, were associated with 22% higher engagement compared to informative posts, and this was due to both higher active and silent engagement.

**TABLE 3:** PICNIC Facebook posts (*n* = 436) characteristics predicting user engagement.

Predictor (post count)	Total engagement	Active engagement (likes/comments/shares)	Silent engagement (consumptions/clicks)
	IRR	IRR	IRR
**Format type**			
Photo (389)	Ref.	Ref.	Ref.
Video (34)	1.18	0.91	1.48
Other (13)	—	0.99	—
**Origin of post**			
Original (385)	Ref.	Ref.	Ref.
Reposted/shared (51)	0.62	0.49	0.84
**Links to health information**			
PICNIC website (77)	0.73	0.79	0.74
Other links (30)	1.36	1.09	1.41
No links (329)	Ref.	Ref.	Ref.
**Communication technique**			
Informative (166)	Ref.	Ref.	Ref.
Instructive (151)	1.22	1.16	1.21
Storytelling (119)	—	—	—
**Answer to question** ^**a**^			
Yes (87)	0.91	0.93	0.92
**Emotion‐inducing**			
Positive (67)	Ref.	Ref.	Ref.
Negative/avoid (52)	0.95	—	—
Supportive (81)	0.86	0.96	0.87
Humorous (97)	—	—	—
Neutral/other (139)	1.08	1.04	1.14
**Age group specific**			
6–12 months (48)	—	—	—
12+ months (100)	1.10	1.08	1.02
Both (288)	Ref.	Ref.	Ref.
**Feeding message** ^**a**^			
Food restriction (55)	1.35	1.18	1.36
Food exposure (137)	1.02	1.07	—
Meal structure (25)	1.14	—	1.11
Meal environment (61)	0.79	0.95	0.74
Food rewards (15)	1.10	—	1.14
Fussy eating (20)	1.40	1.24	1.30
**Type of message** ^**a**^			
Child nutrition (27)	1.16	—	1.03

*Note:* LASSO results for negative binomial regression models predicting the effects of content strategies on engagement of PICNIC Fb posts. Three LASSO analyses were run, one for each of three forms of engagement in this study (total, active, and silent). Best log(lambda) chosen by LASSO and used in the regression was −3.12, −2.68, and −3.11, respectively. The categories prompting engagement; Child health, Recipes/food hacks, and the feeding messages, Pressure to eat, learn to eat, and external influences on feeding were not included in the table as they were all sent to zero (non‐predictive in the analysis). When one variable was selected, all subcategories displayed in the table. Overall categories are in bold while subcategories are below. IRR, which are bolded, have been predicted to increase/decrease engagement more than 10% compared to the reference group.

Abbreviations: IRR, incidence rate ratio; Ref., reference category.

^a^ Present post characteristic compared to not present (reference group).

Feeding messages that focused on food restriction or fussy eating showed the largest positive association with engagement; total engagement was 35% and 40% higher, respectively, compared to the other posts. Meal environment was the only feeding message that had a negative association with engagement. Posts that focused on meal environment were predicted to receive 26% lower silent engagement compared to other posts, although they did not appear to influence active engagement much (IRR 0.95).

### 3.4 Video Views

**FIGURE 2:**
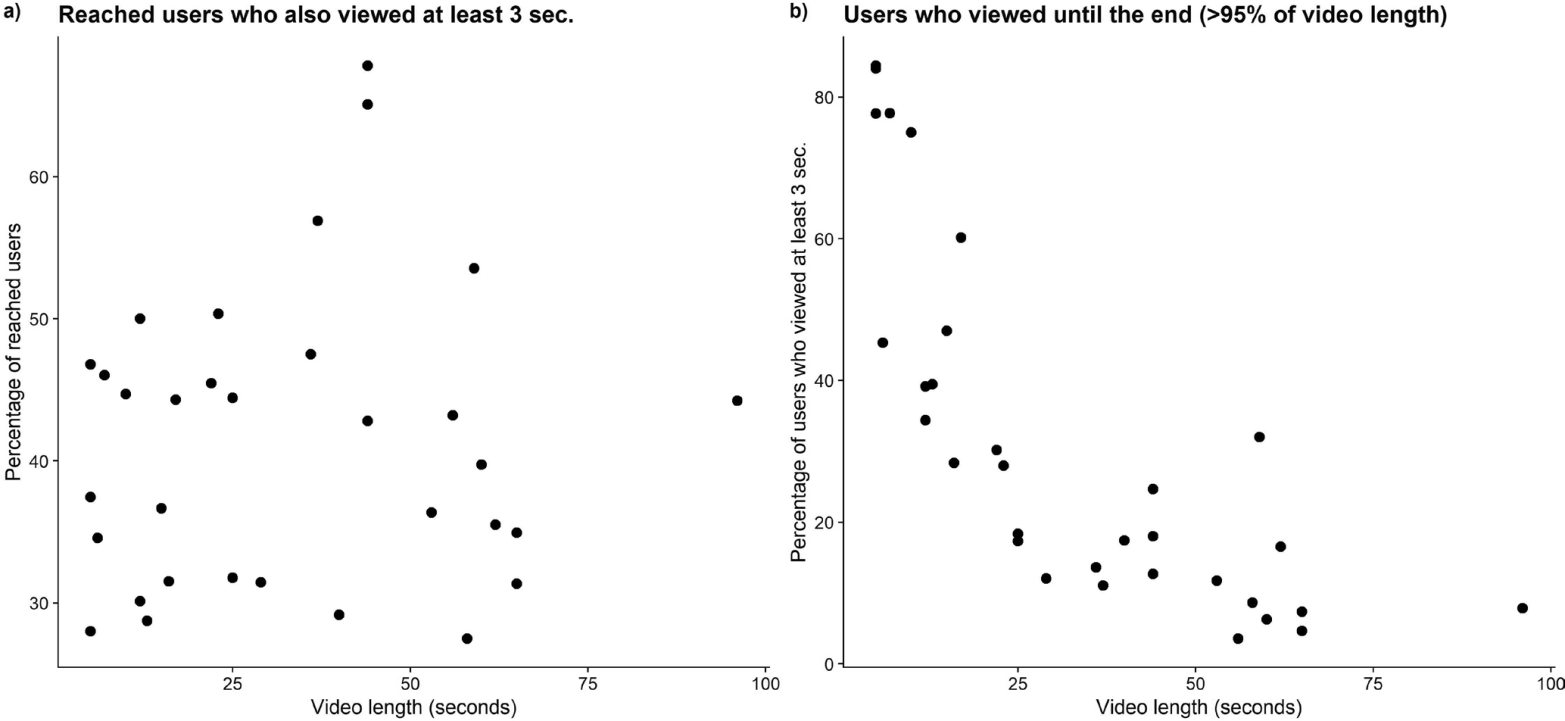
Video views. Scatter plots showing unique views of PICNIC‐produced videos posted on Facebook during the evaluation period. Each post contained intervention (child feeding) messages and were directed towards parents engaged in the PICNIC peer‐education program. (a) Percentage of users who viewed the video (for at least 3 s, as defined by Facebook as “a view”) of all users who were reached by the post. (b) Percentage of the users who also continued to view the entire video (by definition, > 95% of the video length).

PICNIC video posts appeared to attract more silent engagement (i.e., post clicks) than photo posts in our dataset. Silent engagement in this study was defined as post clicks (consumptions) which are measured for both video and photo posts. This includes clicks‐to‐play video views, but it does not include auto‐played video views. The 32 intervention video posts produced by PICNIC during the evaluated period were between 5 and 175 s long and reached between 205 and 921 unique users (median: 355 users). As shown in Figure 2a, typically around 40% (median 41%; IQR 32%–46%) of the reached users stayed to view the video. This corresponds to an average of 212 views per video, of which 93.4% were auto‐played views and 6.6% were click‐to‐play. Figure 2b shows that the percentage of users who continued viewing a video until the end (by definition, > 95% of the video) declined rapidly when videos were longer, with on average 24% of viewers watching until the end when videos were 25 s or longer, compared to 72% for videos that were shorter than 25 s.

## 4 Discussion

Online social networks can be used to disseminate public health interventions (Pretorius et al. 2019), but understanding how to reach and engage target audiences is important (Short et al. 2018). This study found that photo posts reached more users and videos drew more engagement by parents exposed to Fb content from the online PICNIC child feeding program. Original content was associated with higher reach and engagement than reposted or shared posts. Instructive communication techniques and feeding messages about food restriction and fussy eating positively influenced engagement.

Content analyses are not always directly comparable because different coding frameworks, reference groups, and audiences influence results and conclusions (Kite et al. 2019, 2016; Klassen et al. 2018; Barklamb et al. 2020; Rus and Cameron 2016; Hefler et al. 2020). It was therefore important for our study to analyze PICNIC's specific audience to gain relevant insights that can inform future content strategies for parent support.

### 4.1 Videos Associated With Less Reach but More Silent Engagement

The lower reach of video than photo posts contradicted previous research. In an analysis of Australian public health organizations' Fb posts, videos attracted more likes, comments, and shares than photo posts, and about 10 times higher reach. The authors thus speculated that the Fb algorithm preferred videos (Kite et al. 2016). Another study exploring smoking interventions with First Nations people also found that videos had higher reach than other post types, although that relationship did not remain following adjustment for other variables (Hefler et al. 2020). Our findings may differ from other studies due to differences in target audience, number of page followers, and post timing, all of which impact the Fb algorithm. Our approach also differed from previous studies in treating reach as an outcome and a collider, which further limits direct comparisons (Kite et al. 2019, 2016; Klassen et al. 2018; Barklamb et al. 2020; Rus and Cameron 2016). In a content analysis of the six most engaging Fb page posts from popular food industry or lifestyle brands and health promotion organizations, video format was associated with higher active engagement (Klassen et al. 2018), although in a follow‐up LASSO regression analysis including more Fb posts (*n* = 430) the same effect could not be observed (Barklamb et al. 2020). Reach and silent engagement were not explored in those studies.

Higher engagement generally translates into higher reach (Newberry 2023), as was observed in our data. However, despite videos attracting more engagement than photos, their reach was lower; this was possibly due to silent rather than active engagement, which the Fb algorithm prioritizes (Meta 2022). Similar to our findings, a health promotion campaign that adjusted for reach found that organic video Fb posts predicted higher consumption but lower active engagement than photo posts (Kite et al. 2019). In PICNIC, views and post clicks are considered desirable engagement that may lead to behavior change. Parents have previously reported that even a glimpse of previously understood information was an effective reminder of the content (Ball et al. 2022). PICNIC videos were consumed more than was captured by the silent engagement metrics, with 93.4% of the median 212 views per video being auto‐played (i.e., without clicks). Also, the videos were often viewed by 40% of the reached users, which is a large proportion compared to other Australian health promotion pages (Kite et al. 2016), likely reflecting PICNIC content being carefully tailored to the target audience. Videos longer than 25 s had fewer views to completion (Figure 2b). Therefore, we recommend shorter videos and front‐ending the key message to capture interest. Developing videos is labor intensive, so it is difficult to conclude if the increased engagement warrants the time cost, especially considering the potentially decreased reach. It is still unknown how different forms of engagement affect comprehension and retention of knowledge and behavior change. Meta recommends having a storyline to maintain audience interest and states that original content is valued on Fb (Meta 2022). This aligns with our results as original content predicted more reach and engagement than shared content.

### 4.2 Engaging Feeding Messages in Posts

Topics and feeding messages in posts influenced engagement. Posts about food restriction, fussy eating, meal structure, and food rewards were associated with high engagement, and posts about the meal environment had less engagement. It is possible that the overall combination of post characteristics such as format, links, and wording or communication technique influenced silent engagement with these posts, so all these factors need to be considered when planning future posts, especially when aiming to increase engagement with meal environment posts. For example, asking parents what words that relate to meal environment do resonate with them, to draw more engagement to those posts. Another explanation could be that, due to the small number of posts in the categories Meal environment, Role modeling, and Family meals, these were merged for analysis (see File S2). This could have influenced the observed association with meal environment posts, compared to other, more coherent categories.

Notably, using the terms “fussy” or “picky” in posts had the most significant effect on both active and silent engagement. Only 20 posts were coded as “fussy eating” due to PICNIC's intentional effort to normalize children's natural caution of foods (Birch 1998). However, the term is likely relatable because up to 50% of parents perceive their child to be a “picky or fussy eater,” (Carruth et al. 2004; Dubois et al. 2007) In a qualitative content analysis of users' posts on Reddit (another social media platform), fussy eating was found to be a common concern raised by parents of toddlers, who sought support and advice on the platform from peers in similar situations (Fraser et al. 2021). Given parents' interest in “fussy” or “picky” eating, incorporating these terms could serve as an effective strategy to capture initial attention. This initial engagement could be leveraged to segue into other important but potentially less engaging topics, such as food exposure and meal environment.

### 4.3 Instructive Communication Technique Associated With More Engagement

Post engagement is influenced by the communication techniques used. An “instructive” (“how‐to”) communication technique was associated with higher active and silent engagement, showing 22% higher total engagement compared to “informative” content. This finding contrasted previous studies which found that instructive communication had minimal impact (Kite et al. 2019) or negative impact (Kite et al. 2016) on engagement. Differences in coding frameworks and target populations may explain these discrepancies. PICNIC is targeted at parents or carers of young children who are often inundated with parenting information, so may prefer more instructive content. This aligns with a previous study analyzing parental social media posts on fussy eating that revealed a tendency towards seeking practical advice and strategies (Fraser et al. 2021).

### 4.4 Links to PICNIC Website Decrease Post Engagement

Fb's algorithm may reduce the reach of posts containing external links to prevent users from leaving the platform (Cucu 2021). Our analysis showed no association between reach and post links to the PICNIC website; however, posts with external links did have lower engagement than posts with no links. Our previous evaluation study reported that 76% of people who submitted an online expression of interest form to join the program had been directed from social media (Henström et al. 2022). Therefore, external links might still be warranted in PICNIC recruitment posts, but avoided in intervention posts in order to maximize post reach and post engagement.

### 4.5 Strengths and Limitations

This study represents the first content analysis of Fb posts to investigate the effect of content strategies on both reach and engagement of parental feeding messaging. Conducting real‐world translational research, our findings can be applied and tested. Other strengths of this study include iteratively refining the coding framework for consistent coding. Most of the coding was conducted by a female, young adult researcher without dietetics training and therefore similar to the target audience. Unlike previous studies (Klassen et al. 2018; Barklamb et al. 2020; Rus and Cameron 2016; Hefler et al. 2020), we were able to explore the effects of silent engagement (post clicks) as our team had administration access to the PICNIC Fb page data. The project was further strengthened by using LASSO as a model selection tool, which avoids overfitting the data by including a penalty term so that the effect of a predictor is not overestimated (Friedman et al. 2010).

The increase from 1123 to 2067 Fb followers over the 28‐month data collection period might have influenced post performance, so we adjusted for the number of followers in all regression models. Another limitation was our inability to specify what percentage of reach was from the target audience. For example, the PICNIC team answering parents' questions could not be differentiated. This limitation was mitigated by being able to investigate silent engagement, which is not affected by responses to comments. Finally, metrics on engagement and reach do not capture posts or screenshots shared privately as direct messages.

### 4.6 Implications for Research and Practice

Health practitioners communicating with parents about child feeding will benefit from knowing that instructive and original posts, along with those mentioning “fussy” or “picky” eating, have the highest reach and engagement. “Piggybacking” important content in posts that appeal to parents is recommended. Videos may have lower reach but potentially higher engagement in terms of post clicks. These findings could be further influenced by the recent increase in popularity of short videos and reels on social media, which will be interesting to evaluate in terms of usefulness to parents.

Engagement with Fb posts is one of PICNIC's many engagement strategies, so the Fb content is intended to serve as a constant reminder of feeding‐related tips (Ball et al. 2022). It remains unknown how different forms of engagement affect the retention of knowledge and behavior change. Parental interviews may provide further insights about engagement patterns and how different content affects their feeding practices.

It is important for researchers and practitioners to realize that the Fb algorithm is complex, with machine learning used to predict what content will be most relevant to a user (Lada et al. 2021). Social media algorithms constantly evolve, so ongoing monitoring and evaluation of post performance in relevant contexts is necessary. Algorithms should be considered to maximize exposure without compromising content. As the target audience may affect the results of content analyses, our findings are most generalizable to Fb pages targeting parents for nutrition promotion activities aimed at optimizing social media reach and engagement.

Our findings will also be valuable as the PICNIC Project expands to other health districts in Australia, Asia, and as a pilot in Sweden. We recommend that future social media content in PICNIC or similar programs focuses on instructive and original posts and includes terms that parents are most concerned about, such as fussy/picky eating. Future research should prioritize understanding PICNIC parents' perspective on useful child feeding content.

## Author Contributions

**Maria Henström:** conceptualization (equal), data curation (equal), formal analysis (lead), funding acquisition (lead), investigation (equal), methodology (equal), project administration (equal), resources (equal), software (equal), supervision (equal), validation (equal), visualization (lead), writing – original draft (lead), writing – review and editing (lead). **Tayla Esplin:** conceptualization (equal), data curation (equal), formal analysis (equal), investigation (equal), methodology (equal), visualization (equal), writing – original draft (lead), writing – review and editing (supporting). **Emma Schwartzkoff:** conceptualization (equal), data curation (supporting), formal analysis (supporting), investigation (supporting), methodology (supporting), project administration (supporting), supervision (equal), writing – original draft (supporting), writing – review and editing (equal). **Kerith Duncanson:** conceptualization (equal), data curation (supporting), formal analysis (supporting), funding acquisition (supporting), methodology (supporting), project administration (supporting), supervision (equal), writing – review and editing (equal). **Gordana Popovic:** conceptualization (supporting), formal analysis (supporting), investigation (supporting), methodology (supporting), software (supporting), supervision (supporting), validation (supporting), writing – review and editing (supporting). **Richard Ball:** conceptualization (equal), data curation (equal), formal analysis (equal), investigation (lead), methodology (equal), project administration (lead), resources (lead), software (equal), supervision (equal), writing – original draft (supporting), writing – review and editing (equal).

## Ethics Statement

The study has ethics approval from the North Coast New South Wales Human Research Ethics Committee HREC, Ref Number: LNR179, 4/12/17.

## Conflicts of Interest

The authors declare no conflicts of interest.

## Supporting information


File S1. STROBE checklist

File S2. Coding framework

File S3. Examples of PICNIC Facebook posts

File S4. R code for LASSO analyses

## Data Availability

The data that support the findings of this study are available from the corresponding author upon reasonable request. The Rscript produced for the LASSO regression analysis in this study is available in the File S4 of this article.
