# Supplementary material for: Engaging Parents With Child Nutrition and Feeding Information on Facebook: A Retrospective Content Analysis
Source: Food Sci Nutr. 2025 May 25;13(6):e70326. doi: 10.1002/fsn3.70326 (PMC12104198; doi:10.1002/fsn3.70326)
Supplement: Supplementary file 2 — File S2. Coding framework [file FSN3-13-e70326-s001.docx]

**Supplementary Table S2**. Coding framework for organic intervention posts (n=436)

| **Characteristics and codes** | **Description** | **Analysed categories** |
| --- | --- | --- |
| **Format type** | |  |
| Photo/Shared photo | Photo or image with or without text, original i.e., posted by PICNIC admin, or shared/reposted. | Photo (Ref.) |
| Video/Shared video | Video with or without text, original or shared video, or Gif. | Video |
| Link | Link with preview to external source, such as website, with or without caption text. | Other |
| Text only | Text only, Status update with only text, or Update of page profile picture. |  |
| **Origin of post** | |  |
| Original post | Post is uploaded to the platform by PICNIC page admin, i.e., an original post. | Original (Ref.) |
| Reposted/shared post | Uploaded through the platform’s “share” button or an application, that automatically gives credit to the original profile that posted it, or shares a preview link (e.g., to an external website). | Shared |
| **Links to health information** | |  |
| PICNIC website | Clickable link (often in caption text) to somewhere within PICNIC website, can be to the website main page or a specific page within (incl. “about/Expression of interest”). PICNIC logo and non-clickable website addresses is not included. | PICNIC website **^†^** |
| External health information | Links or refer to external health/feeding information that is not on social media, e.g., government website or a third-party website providing health-related information. | Other links |
| Social media profiles | Links to other personal or professional/institutional profiles (or content on those profile pages) on Facebook, YouTube, Twitter, Snapchat, Instagram etc, clickable links or referred to with the use of @ (for example to give credit for a photo). Is often health/feeding related. |  |
| None | No clickable links in the post. | No links (Ref.) |
| **Prompting engagement** ^‡^ | |  |
| Requires answer | Question/Poll/Quiz/Fill-in-the-blank/Game. Direct question as a strategy to get the audience to engage in the post: Specifically asking a question directed to the parents, and/or prompting them to comment on the post in order to answer the question or to share their own experiences/photos: “How does your bub let you know when they're full? Let us know below!”), often in the end of the caption or in the photo; Q&A (asks users to send in questions to be answered). Open/rhetorical questions are not included. | Prompts ^‡^ |
| Requires action | Encourage the user to click on a link (such as referring to the website for more info) or sign-up, asks to share the post, or to tag a friend. |  |
| No prompts | No listed strategies applicable. | None (Ref.) |
| **Communication technique** | |  |
| Informative | Educational posts, providing statistics/facts to inform users on a health issue, its associated behaviours, and/or associated consequences or benefits; informative; can also describe/illustrate a behaviour/consequence (e.g. video of child showing feeding cues, can describe the child’s perspective) but *without* focusing on advice on what to do as a parent; interesting facts, incl. “did you know..?”; research results, infographics, typically more information in the post. | Informative (Ref.) |
| Instructive | ’How-to’ posts (e.g., showing an example of how to do something, how to act in a situation, say or do, can be illustrated in a photo/video or explained in the caption), often bullet-point or clear picture, can be a question or common issue that is answered or “solved” in the post, “instead of…, try…”, or “how to…”, “not helpful vs helpful”, supportive in nature, can be infographics also, recipes, or picture on how to cut a vegetable in a smart/safe way, or example on how to serve or compose a meal. | Instructive |
| Storytelling | Stories of others/Testimonials/Real-life examples/Quotes from parents.  A personal experience/anecdote told, story or quotation, often audience-generated i.e. provided by a PICNIC parent (or made-up but relatable), or shared by PICNIC staff themselves, can also be external/shared story or quote. | Storytelling |
| **Answer to question** | |  |
| Yes / No | The post asks an open or rhetorical question related to feeding, which is then answered in the post. Can also be a post that ‘answers to a frequently asked question’ or a post that answers to a specific question received by a parent. | Yes / No (Ref.) |
| **Emotion-inducing** | |  |
| Positive emotion appeal | Joyful/Happy/Excited/Optimistic/Inspirational/Association with success. Aims to elicit positive emotions like hope and excitement in users, showing success, future health/joy, motivating them to adapt a behaviour for example by providing helpful tips combined with a positive/happy photo and give a feeling of success and food joy. Also includes post that aims to generate a positive feeling about the program (e.g. news on the success/growth of the program). | Positive (Ref.) |
| Avoid/Negative | Aims to elicit negative emotions in users or awareness of foods/behaviours to avoid; warnings; sad examples; avoid this (and why); main focus on the negative aspect or the “wrong feeding behaviour”: avoid this food/doing that, this type of behaviour can result in these negative long-term effects, evoking a feeling of “oh I should avoid that” or “don’t want to end up there”. | Negative |
| Feeling Supported | Aims to address parent anxiety of children’s eating progress their nutrition status; normalising of normal caution of new food; neophobia; normalising slow progress; angst at the dinner table; influencing the eating environment and progress. | Supportive |
| Humorous | Uses any humorous technique (e.g. sarcasm, jokes, catchy pun or memes) to convey a health message, causing laughter and amusement, funny/catchy/cute image, often relatable situation illustrated in a humorous way; or word-playing/Pun – word(s) used in post sound like other words that have different meaning, or catchy/clever, may contain child’ or parents’ made-up speech bubbles; can also be comics/cartoons; makes you smile. | Humorous |
| Neutral/Other | Neutral, or other type of emotion, not able to classify; not applicable. | Neutral |
| **Real-world tie-ins** | |  |
| People | Post contains a quote from, or in another way relates to a celebrity or influencer well known to the audience, such as an entertainment or media profile, a sportsperson, or from a known person of authority or expert in the field. | Not included in analysis **^§^** |
| Events/Culture | Post relates to an event (users may attend) or major holiday; culture (targeting a specific culture, or relates to music, TV-show, travel, or familiar characters from films, children’s books etc), captured at a specific place/location well known to the user; past or current events, Covid-19. |  |
| None | None of the above applicable. |  |
| **Age group specific** | |  |
| 6-12 months | Post content focus specifically on infants up to 12 months. | 6-12 months |
| 12+ months | Post content focus specifically on the age group 12-24 months. | 12+ months |
| Both | Post content is applicable to both age groups, not specific to a particular age. | Both (Ref.) |
| **Feeding message** | | |
| Learn to eat | Refers to reference to learning to eat, when to start feeding solids,  learning new flavours, new textures, chewing gagging/choking, monitoring eating. | Yes / No (Ref.) |
| Division of responsibility; Pressure to eat | Parent provides the food - Child decides to eat or not and how much.  Refers to any type of pressure, persuasion, coercion. Can be something said, facial expressions, direct orders, to eat more, to eat something else within their meal, to stop eating. Refers to the parent interfering with the child’s eating or the child receiving any signal, signs that the parent would like the to do something differently with their eating. | Yes / No (Ref.) |
| Division of responsibility; Food restriction | Parent provides the food - Child decides to eat or not and how much.  Refers to the parent interfering with the child’s eating or the child receiving any signal, signs that the parent would like the to do something differently with their eating. Can be something said, facial expressions, direct orders, to eat more or less, something else within what they are doing, to stop eating. | Yes / No (Ref.) |
| Division of responsibility; Exposure/Introduce new foods | Refers to the parent/carer who decides what food is provided for children.  Strategies for introducing/exposing foods; Number of exposures, new/safe foods, increasing variety; Patience, expectations, gradual progression; Not challenging with foods not yet eaten hence diet is becoming limited; How we serve food, combined, cut, cooked, raw in view of trying out ways bub may be more likely to try a food. Can also refer to post about a child determining what is served either actively or parent responding to what a child eats (safe foods) and only serving that. | Yes / No (Ref.) |
| Division of responsibility; Meal structure | Refers to when and where food is served.  Refers to grazing unstructured eating, adjusting meal timing based on when a child wants to eat. | Yes / No (Ref.) |
| Meal environment/ Role modelling/ Family meals | Refers to creating supportive environment to learn to eat; Eating sitting down, stopping, distractions (intentional/not); Stress/Trauma/fear at table/eating environment; Family attendance behaviour at eating occasions. | Yes / No (Ref.) |
| Food rewards | Refers to using food to achieve an outcome, i.e., to eat, not to eat, behaviour, to soothe, placate, a behaviour, any activity a child. | Yes / No (Ref.) |
| Fussy eating | Refers to parent’s perception of fussiness v normal caution with new food; differing rates at which children progress in eating and variety; the use of generic term which broadly labels children’s eating without further investigation into causes; Picky v problem eating. | Yes / No (Ref.) |
| External Influences on Feeding | Refers to responding to outside influences, situations celebrations, whether with food supply, people, family, grandparents, media, tv, challenges to a parent’s control. | Yes / No (Ref.) |
| **Type of message** | |  |
| Recipes, Food/Serving Advice or Hacks | Refers to recipes or practical advice on how or what to serve, with the post message relating to evidence-based feeding practice advice and knowledge on how children typically learn to eat. This can be posts illustrating how to serve foods or compose meals in ways that increase chances of the child to feel safe and motivated to try the foods (for instance, advice on the amount of food presented to the child, serving foods in separate bowl and not mixed, to serve new foods together with ‘safe’/familiar foods, and whether or not to serve dessert after dinner etc), or picture on how to cut a vegetable in a way that is safe for the child to eat. | Yes / No (Ref.) |
| Child nutrition | Includes general child nutrition information, outside of parental feeding practices. Explicitly mentioning, nutrients or vitamins. | Yes / No (Ref.) |
| Child health related | Includes general child health information, outside of nutrition, e.g., choking risk etc. | Yes / No (Ref.) |

Supplementary Table S2 displays the coding framework used throughout the study. Rules for coding (to ensure consistency); prioritising the message in the photo over the message of the text, the code “Storytelling” over “Instructive” or “Informative”, and the code “Requires answer” over “Requires action”, assign only the most dominant, or two (if equally present), feeding messages provided in the post even if more feeding topics were briefly touched upon. ^†^ External health information, social media profiles and no links were merged into a no/other links group due to small numbers in each subcategory. ^‡^ Requires action and Requires answer were merged into one category “prompts” as there were only small numbers in each subcategory. ^§^ Real world tie ins were not included in the analysis as there were less than 15 posts coded.
