# Supplementary material for: Engaging Parents With Child Nutrition and Feeding Information on Facebook: A Retrospective Content Analysis
Source: Food Sci Nutr. 2025 May 25;13(6):e70326. doi: 10.1002/fsn3.70326 (PMC12104198; doi:10.1002/fsn3.70326)
Supplement: Supplementary file 3 — File S3. Examples of PICNIC Facebook posts [file FSN3-13-e70326-s004.pdf]

Henström M et al. Food Science & Nutrition. 2025

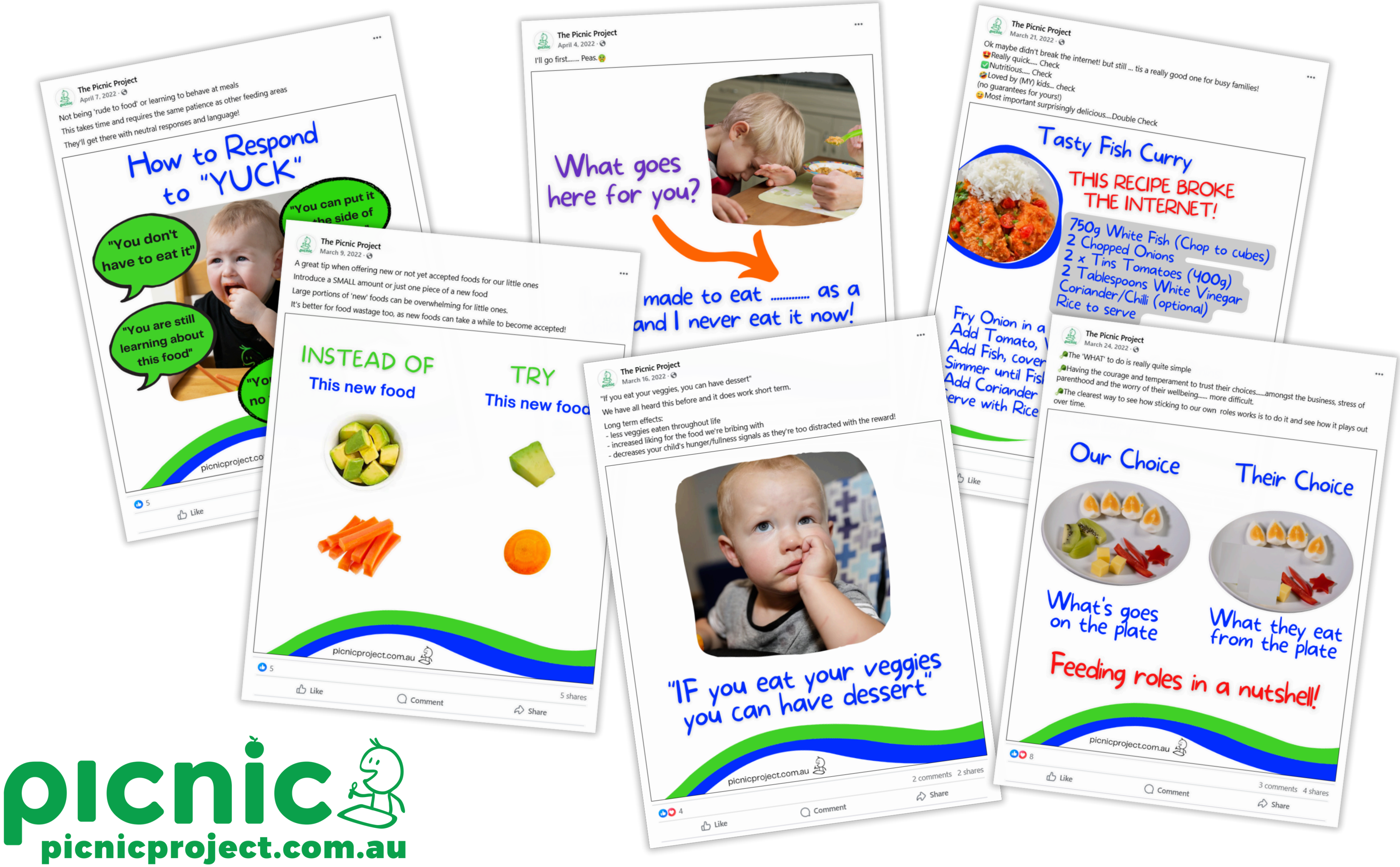

picnic
picnicproject.com.au

Screenshots of some of the intervention posts shared on the PICNIC Facebook page during the evaluation period
