# Supplementary material for: Engaging Parents With Child Nutrition and Feeding Information on Facebook: A Retrospective Content Analysis
Source: Food Sci Nutr. 2025 May 25;13(6):e70326. doi: 10.1002/fsn3.70326 (PMC12104198; doi:10.1002/fsn3.70326)
Supplement: Supplementary file 4 — File S4. R code for LASSO analyses [file FSN3-13-e70326-s003.docx]

##### ******************************************************** ####

# Project: PICNIC content analysis

# Research program: PICNIC (Parents in Child Nutrition Informing Community)

# Project: Content analysis of Facebook PICNIC intervention posts

# Data collection: Jan-Apr 2022, posts posted Jan 2020 - Apr 2022

# R script written by the corresponding author

# ****************************************************

# START UP ####

# ****************************************************

## Install packages (if not already done) ####

install.packages("ggplot2") # for plotting

install.packages("cowplot") # for nicer looking plots

install.packages("tidyverse") # for data management, includes dplyr package

install.packages("psych") # for data management incl descriptives by group

install.packages("MASS") # to run glm neg binomial

install.packages("mpath") # for running negative binomial lasso regression: cv.glmregNB

# ****************************************************

## Load packages ####

library(ggplot2)

library(cowplot)

library(tidyverse)

library(psych)

library(MASS)

library(mpath)

# ****************************************************

## LOAD THE CLEANED DATA ####

# Load the clean R data

load("C:/Users/............/PICNIC_posts_n364.Rdata")

# ****************************************************

## Inspect data ####

# 436 obs (Facebook posts), 32 variables

View(PICNIC)

str(PICNIC)

# Note, factors are already organised with the correct levels for analysis

# to check that there are no missing data:

sum(is.na(PICNIC)) # 0

# Variables in the R dataset ====

# For details, see Table 1 and Supplementary file S2

# Date Date of posting

# Month_posted Month and year of posting

# PostOrganicImpressions Total number of organic post impressions

# PostOrganicReach Total post organic reach

# PostStories_count Total number of active engagement in post (shares + likes + comments)

# share_stories Number of post shares

# like_stories Number of post likes/reactions

# comment_stories Number of comments on the post

# ConsumptionsPost_count Number of post clicks (consumptions)

# TotalEngagement_count Total count of post engagement (active/stories + silent/consumptions)

# PageFollows Number of PICNIC Facebook page followers on the day of posting

# Format_Type Post format: Photo / Other / Video

# Origin Origin of post: Original / Repost/share

# Links Clickabe link used in post: None / Other links / PICNIC web

# Prompt Prompting engagement in post: No prompts / Prompts

# Communication Communication technique used: Informative / Instructive / Storytelling

# Question Post answers to a question: No / Yes

# Emotion Emotion-enducing: Positive / Avoid/Negative / Humorous / None / Supported

# RealWorld Real-world tie-ins (not included in analysis because of two few "yes" <15)

# Age_group Tageted age group: Both / 12+ months / 6-12 months

# Learn_to_eat Feeding message (see Suppl S2 for description); No / Yes

# Pressure Feeding message (see Suppl S2 for description); No / Yes

# Restriction Feeding message (see Suppl S2 for description); No / Yes

# Exposure Feeding message (see Suppl S2 for description); No / Yes

# Structure Feeding message (see Suppl S2 for description); No / Yes

# Environment Feeding message (see Suppl S2 for description); No / Yes

# Rewards Feeding message (see Suppl S2 for description); No / Yes

# Fussy Feeding message (see Suppl S2 for description); No / Yes

# External Feeding message (see Suppl S2 for description); No / Yes

# HacksRecipes Type of message, Recipes/food/serving advice/hacks; No / Yes

# Nutrition Type of message, child nutrition; No / Yes

# Health Type of message, child health related; No / Yes

# ****************************************************

# When plotting, choose what you prefer:

par(mfrow=c(1,2)) # to plot two plots next to each other

par(mfrow=c(1,1)) # to plot one at the time

##### ******************************************************** ####

# Is ENGAGEMENT correlated with REACH? ####

# ****************************************************

## plot of raw data (reach ~ total engagement)

p_tot <- ggplot() +

theme_classic()+

ylab("Reach")+

xlab("Engagement, all types")+

geom_point(data = PICNIC, aes(TotalEngagement_count, PostOrganicReach), size = 0.4)

p_tot

# Looks like a strong trend, but would be better on a log scale.

p_tot +

scale_x_log10() +

scale_y_log10()

## plot of raw data (reach ~ active engagement)

p_act <- ggplot() +

theme_classic()+

ylab("Reach")+

xlab("Engagement, active")+

geom_point(data = PICNIC, aes(PostStories_count, PostOrganicReach), size = 0.4)+

scale_x_log10() +

scale_y_log10()

p_act

## plot of raw data (reach ~ silent engagement)

p_sil <- ggplot() +

theme_classic()+

ylab("Reach")+

xlab("Engagement, silent")+

geom_point(data = PICNIC, aes(ConsumptionsPost_count, PostOrganicReach), size = 0.4)+

scale_x_log10() +

scale_y_log10()

p_sil

# **********************************************

# Spearman correlation (reach~engagement) ====

# it doesn't matter if or how I log-transform since this is non-parametric (rank) test

# Total engagement

# rho correlation coefficient:

cor(PICNIC$TotalEngagement_count, PICNIC$PostOrganicReach, method = "spearman")

# Spearman correlation testing:

Results <- cor.test(PICNIC$TotalEngagement_count,

PICNIC$PostOrganicReach, method = "spearman")

Results

# Active engagement

# rho correlation coefficient:

cor(PICNIC$PostStories_count, PICNIC$PostOrganicReach, method = "spearman")

# Spearman correlation testing:

Results <- cor.test(PICNIC$PostStories_count,

PICNIC$PostOrganicReach, method = "spearman")

Results

# Silent engagement

# rho correlation coefficient:

cor(PICNIC$ConsumptionsPost_count, PICNIC$PostOrganicReach, method = "spearman")

# Spearman correlation testing:

Results <- cor.test(PICNIC$ConsumptionsPost_count,

PICNIC$PostOrganicReach, method = "spearman")

Results

##### ******************************************************** ####

# LASSO REGRESSION ####

# ****************************************************

# Deciding for an appropriate distribution:

# After running diagnostic plots on the different type of regression models, with

# all predictors in the model, including pagefollowers to adjust for growth, we

# decided to use neg binomial regression, which uses log-link.

# That was because the outcome data (reach and engagement) is skewed count data, and

# when running Poisson regression, there was evidence for over-dispersion of data.

# Residual plots looked better with neg binomial regression than other models.

# Most of out predictors are factor variables (only page followers and total engagement are continuous).

# Run LASSO:

# We use 5-fold cross-validation, it is suitable to have as many folds as you have approx

# hundreds of data points (and Barklamb et al used 5-fold as well)

# We will use the entire dataset, not splitting it up on training vs testing data;

# this is because we use LASSO as a variable selection method, to

# be able to see which of our variables are most useful in predicting

# reach or engagement in our dataset.

# We use the package "mpath" to run LASSO with a negative binomial distribution

# and this if the function:

# cv.glmregNB

# default alpha is 1, and this means lasso (i.e., not elastic net regression)

# ****************************************************

# Lasso regression - REACH ####

# ****************************************************

par(mfrow=c(1,1)) # to plot one plot at the time

## > REACH ####

# ****************************************************

# Model with page followers and total engagement (log-transformed) included as predictors

# Set the seed

set.seed(1987)

# Run LASSO

lasso_reach_nb <- cv.glmregNB(PostOrganicReach ~ Origin + Format_Type + Prompt + Links +

PageFollows + log(TotalEngagement_count+1),

data = PICNIC,

nfolds = 5)

# get best lambda

lasso_reach_nb$lambda.optim # 0.07517632

log(0.07517632) # -2.587919

plot(lasso_reach_nb)

abline(v=-2.587919) # added manually, based on the optimal lambda, log(lambda)

# get coefficients for the predictors in the model selected by lasso

round(coef(lasso_reach_nb), 5)

# beta's (coefficients) are then transformed to incidence rate ration (IRR)

exp(-0.12355)

exp(-0.02883)

exp(-0.55581)

exp(-0.07667)

exp(0.04936)

exp(0.04936)

exp(0)

# ****************************************************

# Lasso regression - ENGAGEMENT ####

# ****************************************************

## > Total ENGAGEMENT ####

# ****************************************************

# Set the seed

set.seed(1988)

# Run LASSO:

lasso_totEngage_nb <- cv.glmregNB(TotalEngagement_count ~ Origin + Format_Type + Links + Age_group + Learn_to_eat +

Pressure + Restriction + Exposure + Structure + Environment + Rewards + Fussy + External +

HacksRecipes + Nutrition + Health + Communication + Question + Emotion +

Prompt + PageFollows,

data = PICNIC,

nfolds = 5)

# get best lambda

lasso_totEngage_nb$lambda.optim # 0.04399618

log(0.04399618) # -3.123652

plot(lasso_totEngage_nb)

abline(v=-3.123652) # added manually, based on the optimal lambda, log(lambda)

# get coefficients for the predictors in the model selected by lasso

round(coef(lasso_totEngage_nb), 5)

# all beta's (coefficients) are then transformed to incidence rate ration (IRR)

# (insert the other numbers)

exp(0.33425)

## > Active ENGAGEMENT ####

# ****************************************************

# Set the seed

set.seed(1988)

# Run LASSO:

lasso_actEngage_nb <- cv.glmregNB(PostStories_count ~ Origin + Format_Type + Links + Age_group + Learn_to_eat +

Pressure + Restriction + Exposure + Structure + Environment + Rewards + Fussy + External +

HacksRecipes + Nutrition + Health + Communication + Question + Emotion +

Prompt + PageFollows,

data = PICNIC,

nfolds = 5)

# get best lambda

lasso_actEngage_nb$lambda.optim # 0.06875181

log(0.06875181) # -2.677252

plot(lasso_actEngage_nb)

abline(v=-2.677252) # added manually, based on the optimal lambda, log(lambda)

# get coefficients for the predictors in the model selected by lasso

round(coef(lasso_actEngage_nb), 5)

# all beta's (coefficients) are then transformed to incidence rate ration (IRR)

# (insert the numbers)

exp(0.14607)

## > Silent ENGAGEMENT ####

# ****************************************************

# Set the seed

set.seed(1988)

# Run LASSO:

lasso_silEngage_nb <- cv.glmregNB(ConsumptionsPost_count ~ Origin + Format_Type + Links + Age_group + Learn_to_eat +

Pressure + Restriction + Exposure + Structure + Environment + Rewards + Fussy + External +

HacksRecipes + Nutrition + Health + Communication + Question + Emotion +

Prompt + PageFollows,

data = PICNIC,

nfolds = 5)

# get best lambda

lasso_silEngage_nb$lambda.optim # 0.04475275

log(0.04475275) # -3.106602

plot(lasso_silEngage_nb)

abline(v=-3.106602) # added manually, based on the optimal lambda, log(lambda)

# get coefficients for the predictors in the model selected by lasso

round(coef(lasso_silEngage_nb), 5)

# all beta's (coefficients) are then transformed to incidence rate ration (IRR)

# (insert the numbers)

exp(0.38959)

# **************************************************** ####

## Clear the workspace ####

# ****************************************************

rm(list=ls())

# **************************************************** ####
